# A Fluorescent Probe for Detecting *Mycobacterium tuberculosis* and Identifying Genes Critical for Cell Entry

**DOI:** 10.3389/fmicb.2016.02021

**Published:** 2016-12-20

**Authors:** Dong Yang, Feng Ding, Katsuhiko Mitachi, Michio Kurosu, Richard E. Lee, Ying Kong

**Affiliations:** ^1^Department of Microbiology, Immunology and Biochemistry, University of Tennessee Health Science CenterMemphis, TN, USA; ^2^Department of Pharmaceutical Sciences, University of Tennessee Health Science CenterMemphis, TN, USA; ^3^Chemical Biology and Therapeutics Department, St. Jude Children's Research HospitalMemphis, TN, USA

**Keywords:** *Mycobacterium tuberculosis*, dormant *M. tuberculosis*, fluorescence, imaging

## Abstract

The conventional method for quantitating *Mycobacterium tuberculosis* (*Mtb*) *in vitro* and *in vivo* relies on bacterial colony forming unit (CFU) enumeration on agar plates. Due to the slow growth rate of *Mtb*, it takes 3–6 weeks to observe visible colonies on agar plates. Imaging technologies that are capable of quickly quantitating both active and dormant tubercle bacilli *in vitro* and *in vivo* would accelerate research toward the development of anti-TB chemotherapies and vaccines. We have developed a fluorescent probe that can directly label the *Mtb* cell wall components. The fluorescent probe, designated as DLF-1, has a strong affinity to the D-Ala-D-Ala unit of the late peptidoglycan intermediates in the bacterial cell wall. We demonstrate that DLF-1 is capable of detecting *Mtb* in both the actively replicating and dormant states *in vitro* at 100 nM without inhibiting bacterial growth. The DLF-1 fluorescence signal correlated well with CFU of the labeled bacteria (*R*^2^ = 1 and 0.99 for actively replicating and dormant *Mtb*, respectively). DLF-1 can also quantitate labeled *Mtb* inside of cells. The utility of DLF-1 probe to quantitate *Mtb* was successfully applied to identify genes critical for cell invasion. In conclusion, this novel near infrared imaging probe provides a powerful new tool for enumerating *Mtb* with potential future use in bacterial virulence study.

## Introduction

With more than three deaths per minute, tuberculosis (TB) is a major public health threat. Approximately 9 million new cases of TB are identified per year, with 1.5 million deaths related to TB (WHO, 2015), making TB the greatest cause of mortality due to a bacterial pathogen. The majority of individuals infected with *Mtb* develop latent TB infection (LTBI) where bacteria and the host have established equilibrium without causing apparent symptoms in the host. In LTBI, *Mtb* can persist in host lungs or other tissues for months to decades without replication. Approximately one third of the world population has been latently infected by *Mtb* (Sudre et al., 1992). Because LTBI can be reactivated upon immune suppression, the infected individuals serve as a huge reservoir of active TB in the community. Eliminating latent TB cases would help to reduce active TB significantly.

Research on TB has been impeded by the slow growth rate of *Mycobacterium tuberculosis* (*Mtb*), the major causative pathogen of TB. Conventionally, quantification of *Mtb in vitro* and *in vivo* relies on counting bacterial colony forming units (CFU) on agar plates. *Mtb* divides every 15–20 h. It takes several weeks for *Mtb* to form visible colonies on solid agar plates (Glickman and Jacobs, 2001; Smith, 2003; Zumla et al., 2013). Imaging technologies that are capable of quickly quantitating tubercle bacilli *in vitro* and *in vivo* would greatly accelerate research toward the development of anti-TB chemotherapies and vaccines.

Both bioluminescent and fluorescent protein labeled *Mycobacterium* spp. have been developed for quantitating bacteria in TB research (Andreu et al., 2010, 2012, 2013; Carroll et al., 2010; Kong et al., 2010; Zelmer et al., 2012; Zhang et al., 2012; Ollinger et al., 2013; Singh et al., 2014; Vocat et al., 2015), a number of which have been applied to *in vivo* imaging of *M. tuberculosis* infection (Andreu et al., 2010, 2013; Kong et al., 2010; Zelmer et al., 2012; Zhang et al., 2012). By using non-invasive fluorescent-imaging technologies as laboratory tools to track *Mtb in vitro* and in animals, infections can be visualized in real-time. Although recombinant reporter strains have been used for imaging *Mtb* infection (Andrew and Roberts, 1993; Cooksey et al., 1993; Arain et al., 1996; Hickey et al., 1996; Heuts et al., 2009) *in vitro* and *in vivo*, expression of a foreign gene can impact bacterial fitness (Coulson et al., 1994; Wendland and Bumann, 2002; Rang et al., 2003) or sometimes even be toxic to bacteria (Andreu et al., 2010).

In this study, we developed a fluorescent-imaging strategy that labels macromolecular components of the TB cell wall. This long wavelength fluorescent-imaging probe designated as direct labeling fluorescence-1 (DLF-1), is shown to be highly quantitative. DLF-1 is capable of detecting *Mtb* in both actively replicating and non-replicating dormant states *in vitro*. DLF-1 can also quantitate labeled *Mtb* harbored in cells. In addition, we have successfully verified that DLF-1 can be applied to identify bacterial genes critical for cell entry. This novel imaging probe provides a powerful new tool for quantitating actively replicating and dormant *Mtb in vitro*.

## Materials and methods

### Synthesis of DLF-1

Et_3_N (15.2 mL, 0.109 mmol), oxyma (4.7 mg, 0.0328 mmol), and EDCI (6.3 mg, 0.0328 mmol) were added to a stirred solution of vancomycin (32.5 mg, 0.0219 mmol) and Boc-Gly-OH (4.6 mg, 0.0263 mmol) in DMF (0.2 mL). After being stirred for 22 h at room temperature, the reaction mixture was filtered. The filtrate was purified by reverse phase HPLC to afford Boc-Glycyl-vancomycin (85% yield). 0.1 mL of 4.0 M HCl (dioxane solution) was then added to a stirred solution of Boc-Glycyl-vancomycin (3.9 mg, 2.39 mmol) in dioxane (0.1 mL). After 4 h at room temperature, all volatiles were removed under reduced pressure to afford HCl-Glycyl-vancomycin. Et_3_N (1.0 mL, 7.32 mmol) and cyanin5.5 NHS ester (0.52 mg, 0.732 mmol) in DMF (0.1 mL) were added to a stirred solution of HCl-Glycyl-vancomycin in DMF (0.1 mL). After 6 h at room temperature, all volatiles were removed under reduced pressure to afford the crude DLF-1. This was purified by reverse phase HPLC to afford DLF-1 (>95% purity).

### Verification of DLF-1 binding to PG using isothermal titration calorimetry (ITC)

Binding of bacterial PG peptide to vancomycin or DLF-1 was monitored using an ITC_200_ (Microcal, Piscataway, NJ). Vancomycin hydrochloride from *Streptomyces orientalis* (32 μM final concentration, V2002, Sigma) or DLF-1 (7 μM final) was diluted in 1X PBS pH 7.4 (21-040-CM, Corning), and 204 μL was loaded into the cell. Experiments contained 1% DMSO (BP-231, Fisher) in both syringe and cell, to aid solubility. Peptide Ac-L-Lys-D-Ala-D-Ala was dissolved in the same buffer (600 or 100 μM, respectively), and 39.4 μL was loaded in the syringe. For each binding, three separate experiments were conducted at 25°C (298.15K). Injections were performed with serial injections of peptide; first, one injection of 1 μL, followed by 19 incremental injections of 2 μL, at 120 s intervals. Data from the first injection was excluded, due to pre-equilibration mixing between the contents of cell and syringe at the syringe tip. Peak areas were integrated, normalized, and then fitted by non-linear regression using the independent sites model in Origin™ (version 2.3.6, Microcal, Piscataway, NJ).

### Bacterial growth curves in the presence or absence of DLF-1

The mycobacterium strains (*M. smegmatis, M. bovis* BCG, and *Mtb*) were started from −80°C stocks. For *M. bovis* BCG, and *Mtb*, cultures growth in the presence or absence of DLF-1 were performed in 7H9 broth (Difco, Detroit, MI) supplemented with 0.5% glycerol, 10% oleic acid dextrose complex without catalase and 0.05% Tween 80 (MOAD/T medium) at 37°C without shaking. The bacterial cultures were then started in the presence of different concentrations of DLF-1 (0, 100 nM, 1 μM) and monitored OD over time in a 96-well plate with a Tecan multimode reader (Infinite 200 Pro, Tecan) at absorbance 600 nm with bandwidth 9 nm and number of flashes as 25. OD was measured daily for *M. bovis BCG* and *Mtb* to plot growth curves. For *M. smegmatis*, culture was set up in a 96-well plate, and the plate was kept in the reader at 37°C. OD was measured hourly with automatic shaking for 10 min before OD measurement. The Supplementary Figure 1 shows the correlation between the OD values measured with the Tecan microplate reader at 600 nm and the OD value in a cuvette measured with commonly used ThermoScientific Genesys 20 spectrophotometer (ThermoFisher Scientific) at 600 nm, which helps convert the OD values of the Tecan microplate reader into OD values in a cuvette measured with the Genesys 20.

### MIC of DLF-1 against *M. smegmatis*

Minimum inhibitory concentration of DLF-1 against *M. smegmatis* at which 90% of strains were inhibited (MIC_90_) was determined using the standard broth microdilution method in 96-well microtiter plates as described previously with minor modifications (Swenson et al., 1982). Briefly, serial doubling dilutions of DLF-1 were prepared in MOAD broth to achieve final concentrations ranging from 8 to 0.125 μg/ml. *M. smegmatis* was added to each well with a final inoculum of ~10^5^ CFU/ml. The plate was incubated at 37°C. OD_600_ was measured at 48 h with a Tecan microplate reader. The wells with *M. smegmatis* alone were controls.

### Direct labeling bacteria with DLF-1

Bacterial cultures were stopped when they reached the logarithmic growth phase (OD_600_, 0.4~0.6). For each strain of studied bacteria, a series of dilutions were made starting from OD_600_ of 1.0 measured with Genesys 20 in growth medium. Bacteria cells were centrifuged at 9000 rpm for 5 min, and cell pellets were washed with 1X PBS twice and finally resuspended in 1X PBS containing 100 nM DLF-1 for 3 h on ice (for fast-growing *M. smegmatis)* or at room temperature (for *M. bovis BCG* and *Mtb* CDC1551). After incubation with DLF-1, bacterial cells were centrifuged (9000 rpm, 5 min), washed once with 1X PBS (10,000 rpm, 10 min) to remove unbound DLF-1, and were finally resuspended in equal volumes of 1X PBS solutions. Each sample was added to a fluorescence 96-well microplate, and fluorescence intensity of DLF-1 was measured with a multimode fluorescence plate reader (Infinite^R^ 200 PRO, Tecan) at excitation 680 nm and emission 710 nm. Each strain of bacteria was also titrated to enumerate CFU. For determination of threshold of detection, we optimized the condition of the Tecan reader, with gain value as 150, excitation 670 nm and emission 700 nm. For cell infection, a series of concentrations of a tdTomato-expressing *Mtb* strain were incubated with 100 nM DLF-1, then were used to infect THP-1 macrophages and A549 human lung epithelial cells. Fluorescence was measured 3-h post infection by the Tecan plate reader.

### Fluorescent microscopy of DLF-1 labeled *Mtb* inside cells

A tdTomato expressing *Mtb* strain (Erdman-pJDC60) was applied to infect cells after labeling bacteria with DLF-1. This strain was cultured in MOAD/T medium with addition of 25 ug/ml kanamycin until it reaches the logarithmic phase (OD_600_, 0.4~0.6). A549, or THP-1 cells were grown on the coverslips in 24-well cell culture plates at a concentration of 2 × 10^5^ cells/well. For THP-1, phorbol 12-myristate 13-acetate (PMA) was used to stimulate the cells for 3 days. The cells were then infected with bacteria at 37°C for 3 h. After bacterial infection, extracellular bacteria were aspirated, and the infected cells were washed with 1X PBS, treated with amikacin (200 μg/ml) for additional 2 h to eliminate extracellular bacteria as described previously (Mehta et al., 1996; Napier et al., 2011). Bacterium infected cells were then fixed with 4% freshly made paraformaldehyde for 10 min at room temperature. After washing with 1X PBS for three times, the fixed infected cells were permeabilized with 0.2% Tween-80/PBS (v/v) solution for 5 min, washed again with 1X PBS for three times, and then stained with DAPI solution (300 nM) for 5 min in the dark. After another three-time washing with 1X PBS, the coverslips containing bacterium infected cells were taken out and mounted to glass slides for fluorescence microscopy analysis with a deconvolution microscope (Carl Zeiss). For imaging of DLF-1 labeled dormant *Mtb* inside macrophages, a *Mtb* CDC1551 strain carrying a genome integrated tdTomato gene (CDC1551-pYK13) was cultured in a modified Wayne non-replicating persistence model. THP-1 cells seeded in a 24-well plate containing cover slips were stimulated with PMA for 3 days, and then infected with the DLF-1 (100 nM) labeled, non-replicating td-Tomato-expressing *Mtb*. After bacterial infection, the same protocols of DAPI staining, slide mounting, and microscopy imaging were conducted. Images were taken with a 63X oil lens.

### Construction of a *Mtb* strain carrying a genome-integrated tdTomato gene

We constructed a tdTomato-expressing *Mtb* in a more stable manner by site-specifically integrating a single copy tdTomato into the mycobacterial chromosome, using a mycobacterial plasmid containing the phage attachment site *attP* and the L5 integrase (*int*) gene from the mycobacteriophage L5 genome, as has been shown by others for bioluminescent reporters (Andreu et al., 2010, 2013). To construct this vector, we began with replacing the GFP gene in pFJS8, an *E. coli*-mycobacterium shuttle plasmid containing the L5 promoter (Miltner et al., 2005), with a fragment from pDONR222 (Invitrogen) containing a suicide *ccdB* gene and a chloramphenicol resistance gene (Cm^R^) at restriction sites of *Hind*III/*Kpn*I. In this way, the expressions of *ccdB* and Cm^R^ are under a L5 promoter. We then cut the L5-*ccdB*- Cm^R^ fragment from the new vector at *Xho*I and *Kpn*I and ligated it into *Xho*I/*Kpn*I digested pYUB412, a mycobacterial integrating cosmid vector (Bange et al., 1999). The tdTomato gene was then PCR amplified from pRSETB-tdTomato using an up-stream primer containing *Hind*III site at 5′ end and a down-stream primer containing *Kpn*I site at 5′ end (F: 5′-TATAAAGCTTGGATCCATGGTGAGCAAGG-3′; R: 5′-TATAGGTACCGAATTCTTACTTGTACAGCTCGTCC-3′). The purified PCR product was then digested with *Hind*III and *Kpn*I and ligated into the *Hind*III/*Kpn*I digested pYUB412 containing L5-*ccdB*- Cm^R^. The new plasmid is designated as pYK13. After transformation of the pYK13 into CDC1551, we selected colonies with hygromycin selective agar plates, and confirmed the success of integration of tdTomato into *Mtb* genome by PCR amplifying the tdTomato and hygromycin resistance genes, and by the presence of tdTomato specific fluorescence.

### Non-replicating persistence model

We modified the Wayne non-replicating persistence (hypoxic shift-down) model by substituting Dubos with 7H9 (containing 0.05% Tween 80 and 10% OAD) and using the *Mtb* strain expressing genome-integrated tdTomato (CDC1551-pYK13). *Mtb* cultures were set up at 10^5^–10^6^ cfu/ml in 12 × 75 mm tubes in a total volume of 3.0 mL (head space ratio, HSR = 0.5). Each tube contained a 13 × 4 mm diameter stir bar (Fisher, PA, USA). Cultures were incubated in a 37°C incubator on a stir plate with 200 rpm. Some cultures also contained Methylene Blue at a concentration of 1.5 mg/L to be used as an indicator of oxygen depletion. An additional tube of Methylene Blue without *Mtb* was used as a color reference. When the Methylene Blue-containing tube turned colorless, cultures were incubated for an additional week (total 18–22 days), and then were used for DLF-1 fluorescence labeling. A sample of the culture was carefully removed from the middle portion of each tube by pipette to avoid disturbing the pellicle on the surface or the sediment on the bottom. Optical density reading (600 nm) of the cultures was determined. A series of dilutions were made and then centrifuged (9000 rpm, 5 min), washed once with 1X PBS/T, and then resuspended in 1X PBS. DLF-1 labeling of the dormant cultures were performed similarly as for labeling of other bacteria before. The *Mtb* CDC1551 strain carrying a genome-integrated tdTomato gene was cultured in the Wayne model to reach dormant state, diluted in various concentrations, and then labeled with DLF-1. Fluorescent intensity of DLF-1 labeled dormant *Mtb* was plotted against the concentration of bacteria or concentration of DLF-1, respectively. All conditions were set up in triplicates. Samples of dilutions were taken for CFU enumeration on 7H10 agar plates in duplicates. Colonies were enumerated at 4–6 weeks following inoculation.

### Using DLF-1 labeling to identify genes important for cell invasion

*Mtb* transposon mutant of *plcC*, CDC1551, Δ*hbhA* and MT103 strains were cultured in MOAD/T medium. Bacterial cells grown to middle-logarithmic phase (OD_600nm_, 0.4~0.6) were harvested, labeled with 100 nM DLF-1 at room temperature for 3 h, washed and suspended in antibiotic free A549 cell culture medium. A549 cells were cultured and seeded to 96-well fluorescent microplates (5 × 10^4^/well) 1 day before experiment. DLF-1 labeled *Mtb* mutants and wild type control strains were used to infect A549 cells at a MOI of 50. Fluorescence of the microplates was measured to quantitate bacteria loaded initially. After 3 h of infection, infected cells were washed once with 1X PBS, then added cell culture medium containing 200 μg/ml amikacin and incubated for another 2 h to remove extracellular bacteria. After aspirating amikacin containing medium and washed with PBS solution, fluorescence of the microplates were measured to quantitate intracellular bacteria. Bacterial infection rate of each strain was calculated as the average fluorescence intensity at the end of infection divided by the average fluorescence at the beginning. Bacterial infection ratio of mutant to wild type control is calculated as the mutant infection rate divided by that of wild type strain. Bacterial samples from the initial and end of infection were taken and plated for CFU analysis.

## Results

### DLF-1 is a high affinity stoichiometric probe for the D-Ala-D-Ala motif of bacterial peptidoglycan (PG)

**Figure 1:**
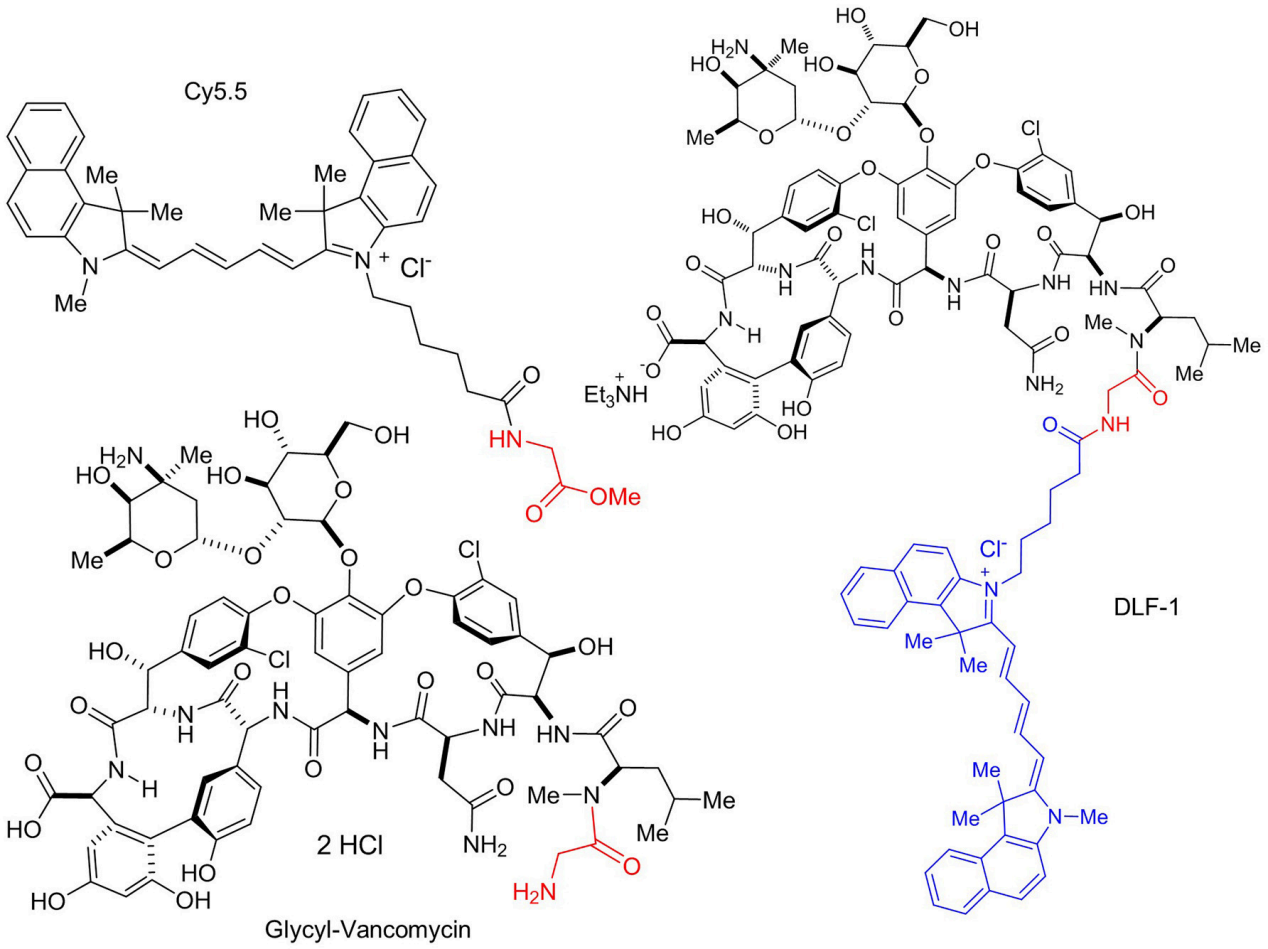
**Structure of DLF-1**. DLF-1 is synthesized by conjugating glycyl-vancomycin with a near infrared dye Cy5.5.

**Figure 2:**
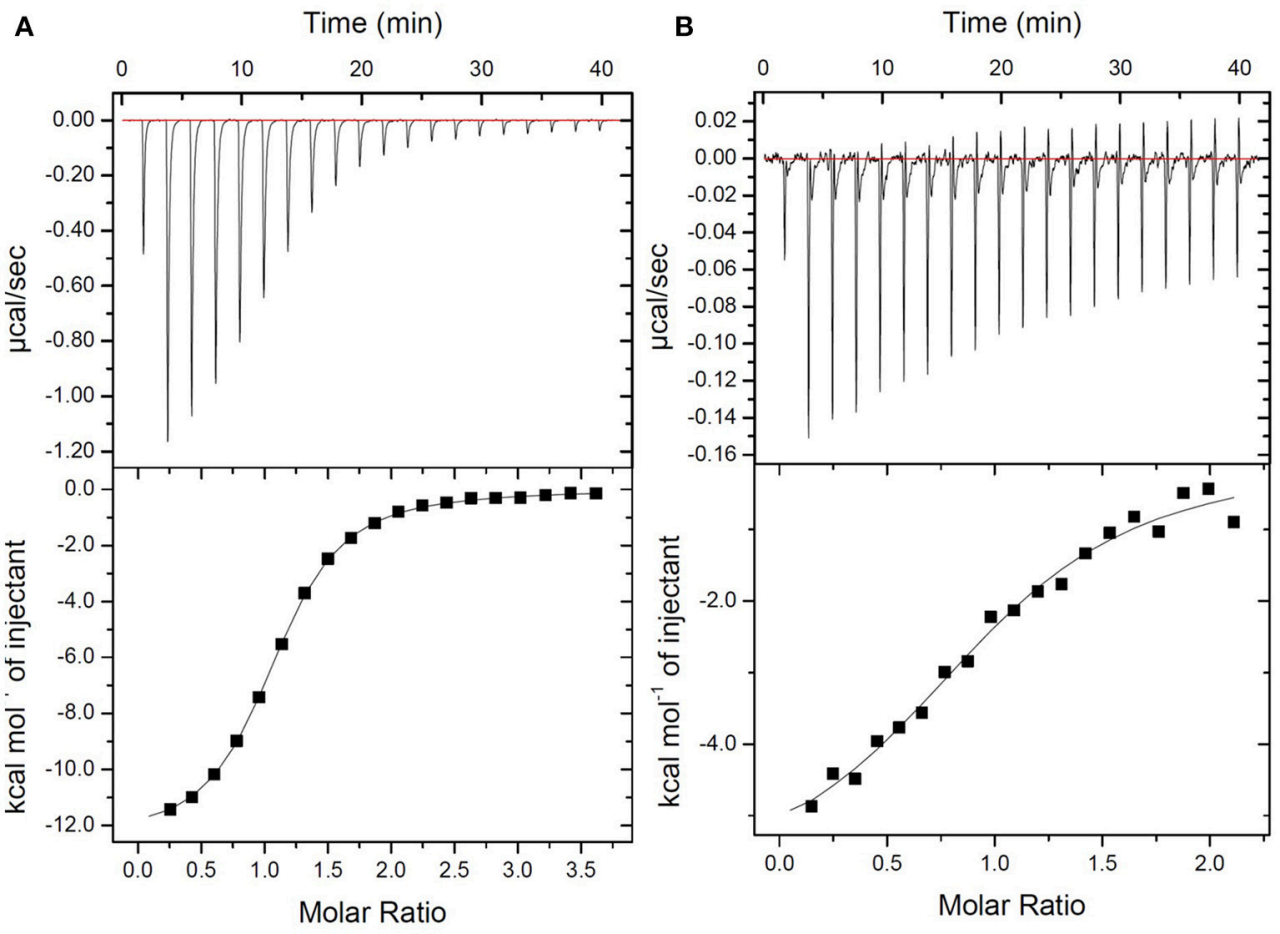
**Thermodynamic evaluation of Ac-L-Lys-D-Ala-D-Ala-OH binding to DLF-1**. Top: Raw curve of power vs. time. Bottom: Fit of integrated peak areas (heat released per injection) used to determine the thermodynamic parameters presented in Supplementary Table 1. **(A)** Representative ITC data measuring the thermodynamics of binding of peptide to Vancomycin. Peptide (600 μM) was titrated into a solution containing vancomycin (32 μM) in 1X PBS at 25°C. **(B)** Representative ITC data measuring the thermodynamics of binding of peptide to DLF-1. One percent of DMSO was added to control and test titrations, as it increased solubility of DLF-1.

The new fluorescent probe was designed to bind to the terminal peptide D-Ala-D-Ala of the PG precursor. DLF-1 was synthesized by conjugating fluorescent Cy5.5 with glycyl-vancomycin (Figure 1). Isothermal Titration Calorimetry (ITC) was applied to verify that DLF-1 binds to the D-Ala-D-Ala motif of PG. ITC measures the heat release (or absorption) associated with a binding event, offering an orthogonal, non-fluorescence based, validation of binding (Pierce et al., 1999). ITC shows that DLF-1 binds stoichiometrically (*N* = 0.99) to the peptide Ac-L-Lys-D-Ala-D-Ala (AcKAA) in PBS (Figure 2) with high affinity (K_d_ 2.3 μM). The binding affinity for DLF-1 is similar to parent molecule vancomycin (2.5 μM, Supplementary Table 1) and in line with prior studies (Leavitt and Freire, 2001; Rekharsky et al., 2006). These results suggest that DLF-1 has suitable biochemical target binding properties to advance to microbial profiling.

### DLF-1 does not inhibit growth of bacteria at the working concentration

**Figure 3:**
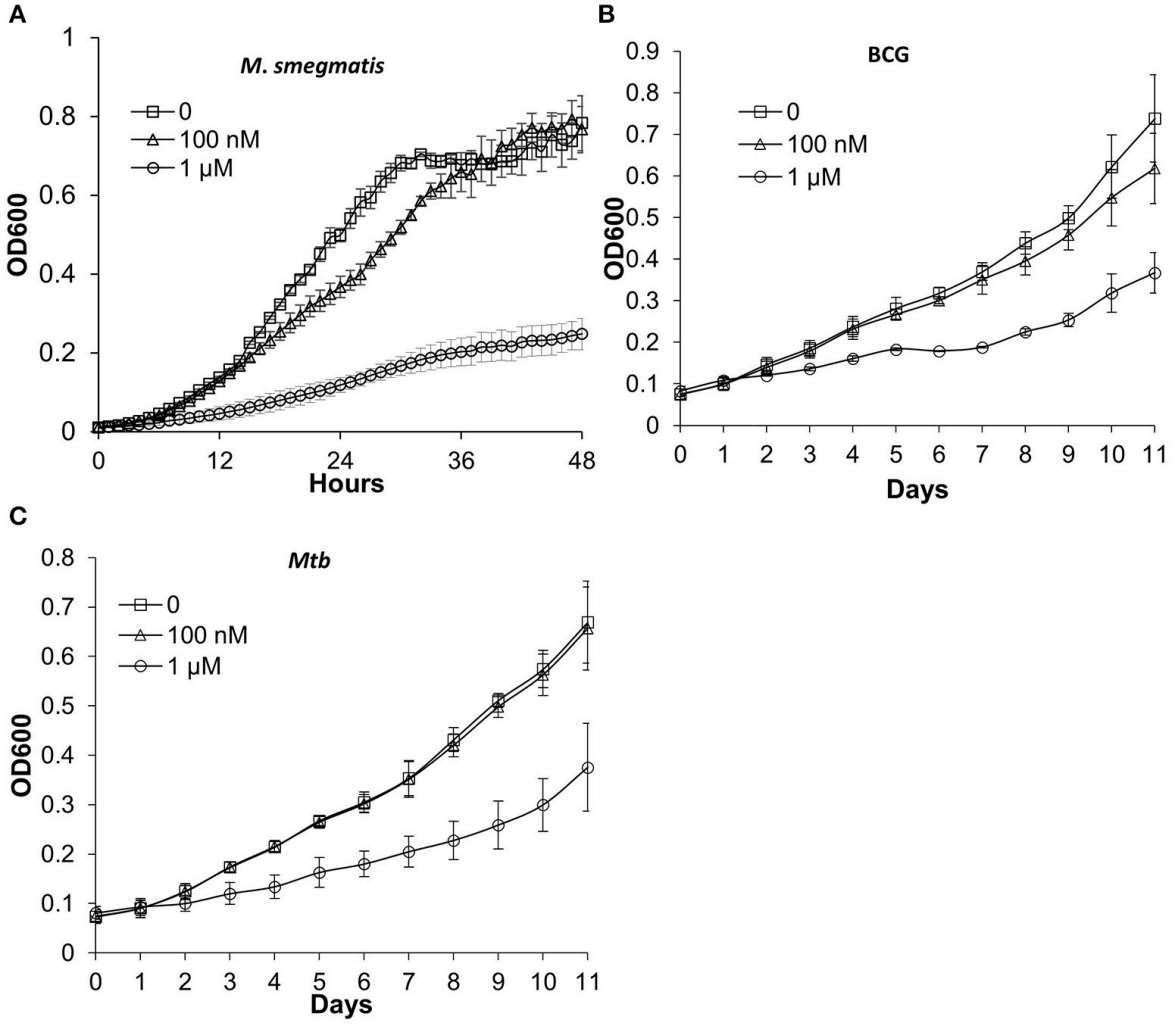
**Mycobacterial growth curves in the presence or absence of DLF-1. (A–C)** represent the growth curve of each mycobacterium in media containing 0, 100 nM, 1 μM of DLF-1, respectively. **(A)** *Mycobacterium smegmatis*; **(B)** *Mycobacterium bovis* BCG; **(C)** *Mycobacterium tuberculosis* CDC1551. Error bars represent standard errors of means calculated based on results from three independent experiments.

In order to label actively growing bacteria, chemical probes should not affect bacterial growth. We tested whether DLF-1 labeling inhibits growths of *M. smegmatis, M. bovis* bacillus Calmette-Guérin (BCG) and *Mtb* CDC1551. Two concentrations of DLF-1 were used to incubate with bacteria grown in liquid media. The growth curves of bacteria incubated with two concentrations of DLF-1 were compared with those without DLF-1. One μM of DLF-1 in culture media significantly inhibited growth of *M. smegmatis*, while having much less of effect on growth of *M. bovis* BCG and *Mtb* CDC1551. When 100 nM DLF-1 was used, no significant effect on growth was observed for *Mtb* (Figure 3). The MIC_90_ for DLF-1 against *M. smegmatis* was determined as 1 μg/ml (~0.45 μM) by broth microdilution method. We then decided to use 100 nM of DLF-1 for the remaining bacterial labeling.

### DLF-1 can be used to accurately quantitate mycobacteria *in vitro*

**Figure 4:**
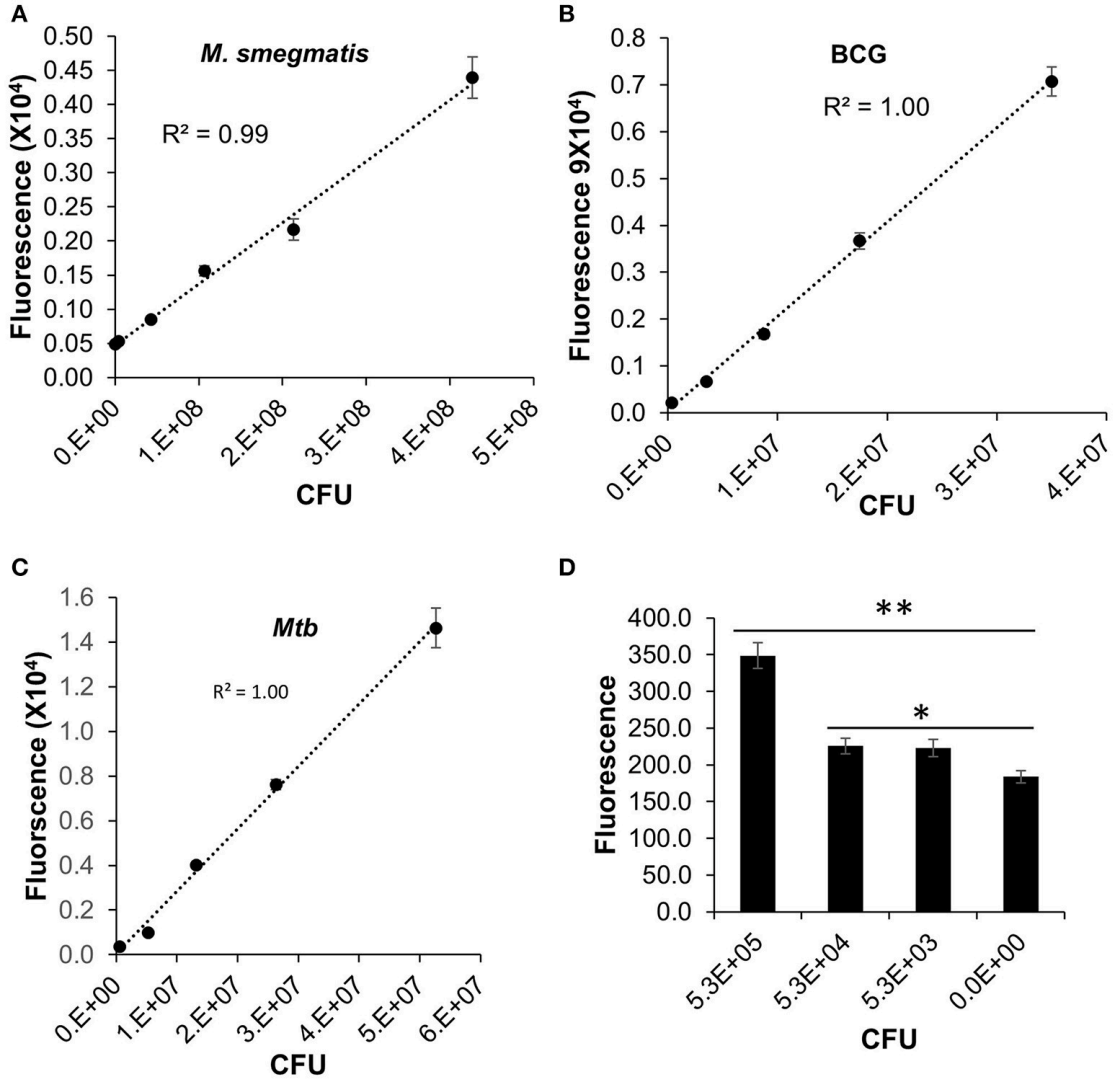
**Direct labeling of different mycobacteria with DLF-1**. Fluorescent intensities of DLF-1 labeled bacteria were plotted against the numbers of bacteria. **(A**–**C)** represent plots for *M. Smegmatis, M. bovis* BCG, and *Mtb* CDC1551, respectively. Linear trend lines with R-squares are also displayed for each plot. Linear range: *M*. *smegmatis* 4.3 × 10^5^−4.3 × 10^7^; *M. bovi*s BCG 3.5 × 10^4^−3.5 × 10^7^; and *Mtb* 5.3 × 10^4^−5.3 × 10^7^. **(D)** Minimum number of *Mtb* within the detectable linear range. ^**^P < 0.01; ^*^P < 0.05. Error bars represent standard errors of means calculated based on results from three independent experiments.

We have validated the application of DLF-1 to detection of actively replicating *Mtb in vitro*, in comparison with fast-growing *M. smegmatis*, and slow-growing *M. bovis* BCG. The correlation between fluorescent signal and bacterial numbers was evaluated after DLF-1 labeling of each *Mycobacterium* spp. Fluorescence generated from DLF-1 labeling linearly correlated with bacterial CFUs in all tested mycobacterial strains (Figure 4). The lowest number of *Mtb* within the linear range is 5.3 × 10^4^ (*P* < 0.05, Figure 4D).

We also examined the threshold for differentiation of DLF-1 labeled *Mtb* from the same number of *Mtb* without DLF-1 labeling using an optimal combination of excitation and emission wavelengths and gain value set up in the multimode reader. An *Mtb* strain carrying a plasmid expressing tdTomato was used (Kong et al., 2016), and the tdTomato fluorescence served as an internal control for DLF-1 labeling. When measured at the optimal excitation and emission wavelengths of the Cy5.5 fluorophore on DLF-1, DLF-1 labeled *Mtb* showed significantly higher fluorescence than the bacteria without labeling at each dilution of bacteria (Supplementary Figure 2A). When measured at the appropriate wavelengths for tdTomato, there was no such a difference between the two groups at any dilution of bacteria, suggesting there are the same bacterial numbers in the two groups at each dilution (Supplementary Figure 2B). The threshold of detection of *Mtb* for DLF-1 is ~10^3^ CFU using the multimode plate reader with the optimal wavelengths and gain value described in Materials and Methods (*P* < 0.05). Although this condition can differentiate 10^3^ CFU from the non-labeled control, the detected fluorescence is not correlated with CFU very well (*R*^2^ = 0.75).

### DLF-1 labeling can quantitate *Mtb* inside of cells

**Figure 5:**
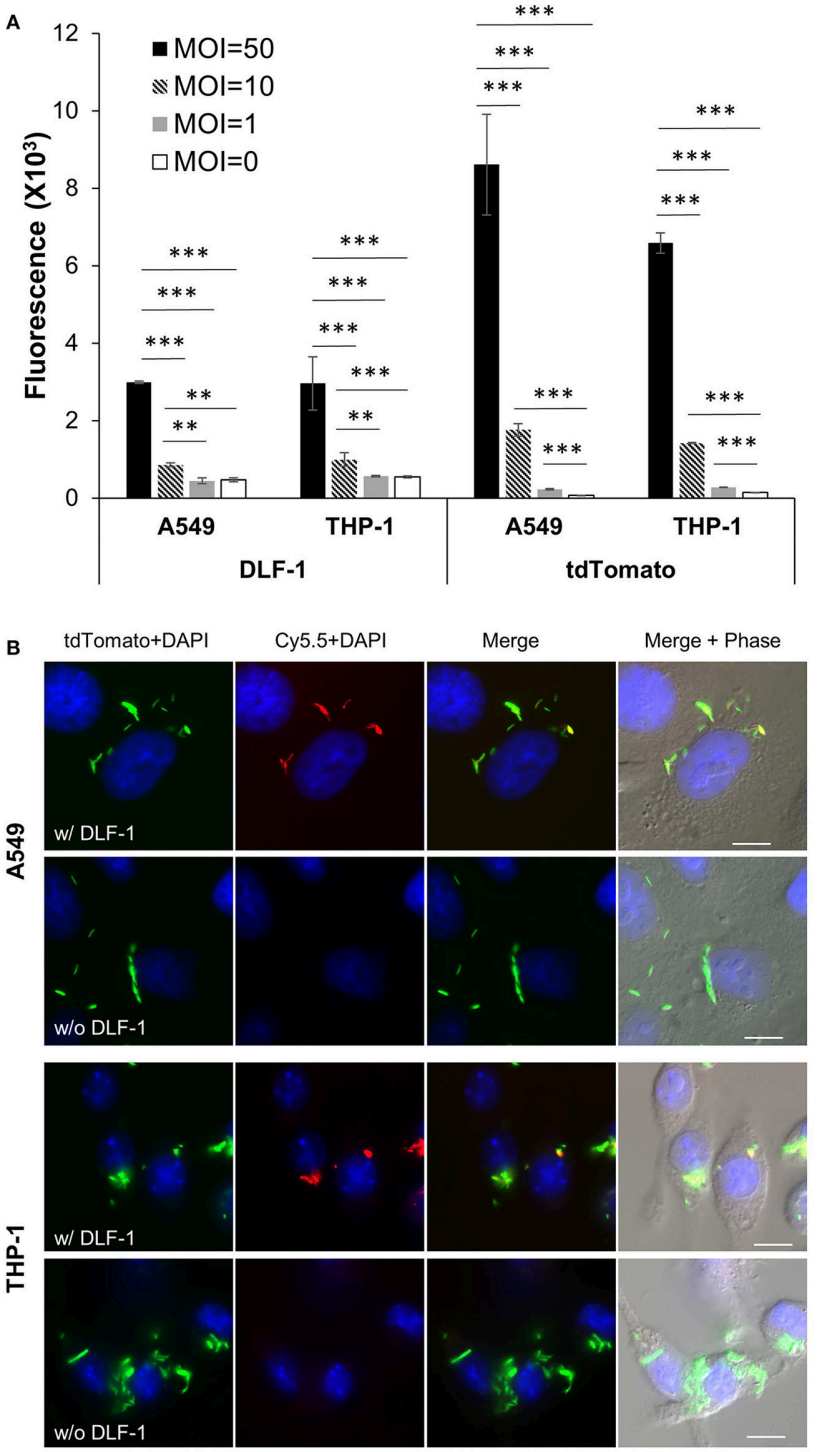
**Imaging infection of human epithelial cell line (A549) and human macrophage cell line (THP-1) with DLF-1 (100 nM) labeled *Mtb***. **(A)** Fluorescence detected from cells infected with DLF-1 labeled *Mtb* at different MOIs *in vitro*. Fluorescent intensities were measured for the labeled DLF-1 (Cy5.5) and for the endogenous fluorescent tdTomato protein, respectively. Error bars represent standard errors of means calculated based on results from three independent experiments. One-way ANOVA test was conducted for assessing overall differences among groups, and Turkey's multiple comparison tests were applied to assess differences between two groups. ^**^P < 0.01; and ^***^P < 0.001. **(B)** Images of DLF-1 labeled *Mtb* inside of cells. THP-1 and A549 were infected with a tdTomato-expressing *Mtb* strain which has been labeled with DLF-1. Red, pseudo-colored DLF-1; Green, pseudo-colored tdTomato; and Blue, DAPI. White scale bars represent 10 μm.

We examined if DLF-1 could be applied to image *Mtb* in cell infection models. A tdTomato-expressing *Mtb* strain was incubated with DLF-1 and infected THP-1 macrophages and A549 human lung epithelial cells. Fluorescence was detected from MOI = 10 in both cell lines (Figure 5A), and the fluorescence measured from DLF-1 labeled *Mtb* increased as MOIs rose. The fluorescent intensity of endogenous tdTomato among different MOI groups in A549 or THP-1 cells was measured in parallel with fluorescence of DLF-1. Cell infection with DLF-1 labeled *Mtb* was further verified by fluorescent microscopy. Slides with fixed infected cells were imaged with a fluorescent deconvolution microscope using pseudo colors: tdTomato-green, Cy5.5-red and DAPI-blue. As shown in Figure 5B, tdTomato and Cy5.5 are co-localized in cytoplasm of THP-1 and A549, which confirmed that DLF-1 can be applied to detect labeled *Mtb* inside of cells.

### DLF-1 can label non-replicating dormant *Mtb in vitro*

In order to study molecular mechanisms involved in the onset of latency and/or reactivation, several *in vitro* and *in vivo* models have been developed to mimic LTBI in human. The Wayne model developed in 1996 is a well-characterized *in vitro* model based on hypoxic growth of *Mtb* (Wayne and Hayes, 1996). In this model, tubercle bacilli are cultured in an undisturbed liquid medium and exposed to oxygen depletion by a way of a completely sealed culture container. *Mtb* undergo an orderly process of metabolic shutdown, and develop resistance to rifampicin and isoniazid, but are sensitive to the anaerobic bactericide metronidazole (Wayne and Lin, 1982; Wayne, 1994; Wayne and Hayes, 1996). To validate DLF-1 in detecting dormant *Mtb*, we labeled dormant *Mtb* generated by the Wayne model with DLF-1.

An *Mtb* CDC1551 strain with a genome-integrated tdTomato gene was used in the Wayne model to generate dormant *Mtb*. With this strain, fluorescence of tdTomato can serve as an internal positive control for DLF-1 labeling. The dormant state of *Mtb* from this model was confirmed by verifying drug tolerance. After *Mtb* reached dormant state, 0.1 μg/ml rifampin was added in the culture for 3 days. The proportion of survived dormant *Mtb* reached 23.5 ± 4.8% (calculated as CFU of treated/CFU of untreated), which is consistent with previously reported results (Wayne and Hayes, 1996). With the same dose of rifampin, 97.5% of actively replicating *Mtb* was killed (Supplementary Table 2).

**Figure 6:**
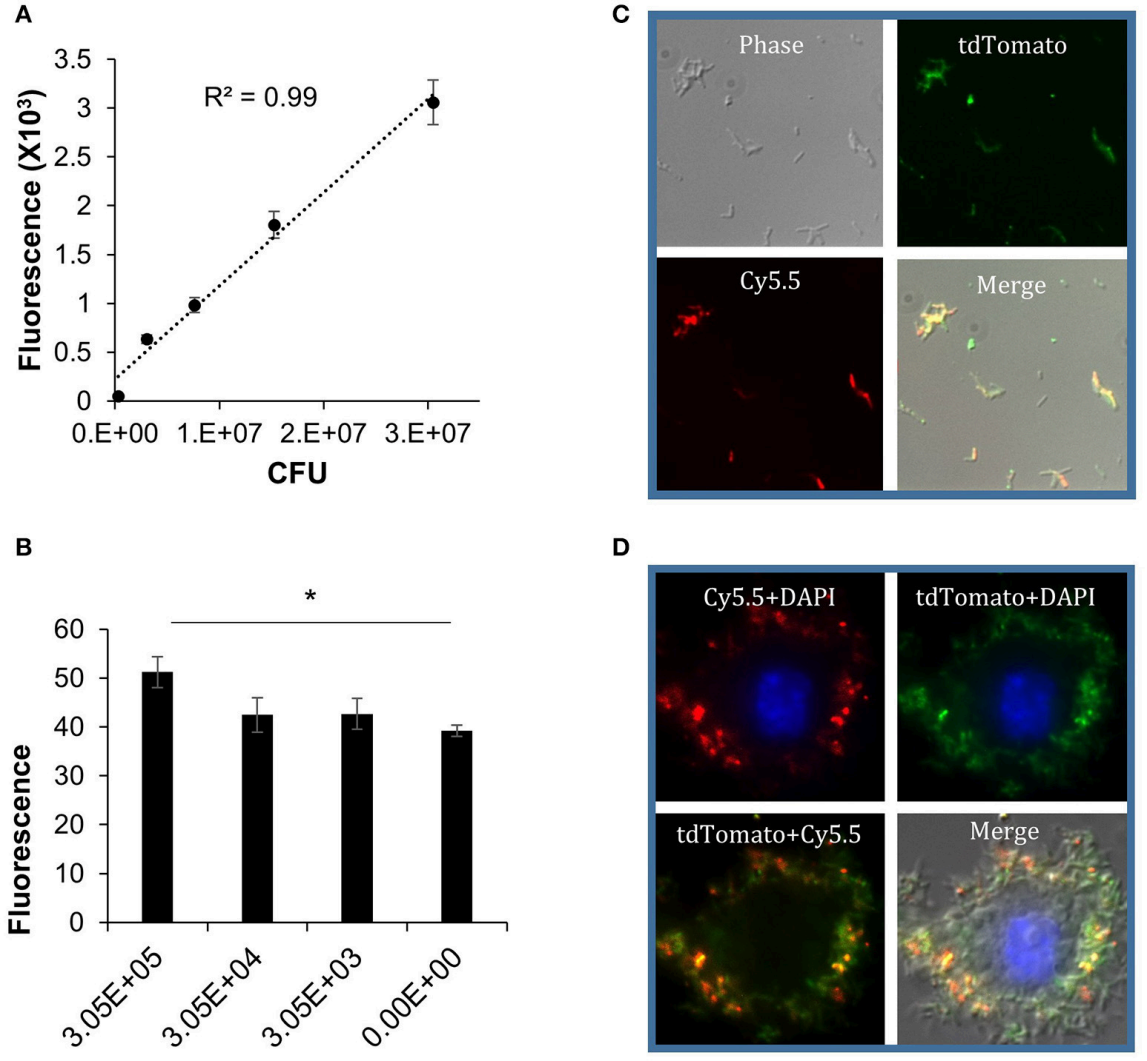
**DLF-1 labeling of dormant *Mtb* cultured in Wayne model**. **(A)** Correlation between fluorescence measured with DLF-1 wavelengths and bacterial numbers of dormant *Mtb*. *Mtb* was labeled with DLF-1 at 100 nM. **(B)** Threshold detection of DLF-1 labeling dormant *Mtb* in culture. ^*^P < 0.05. **(C)** DLF-1 (100 nM) labeling dormant *Mtb* smeared on slides, and imaged with a fluorescent microscope. **(D)** Microscopy images of the DLF-1 (100 nM) labeled dormant *Mtb* strain carrying a genome-integrated tdTomato gene inside of THP-1 macrophages. Red, Cy5.5; Green, tdTomato; Blue, DAPI stained nuclei.

After the hypoxic dormant bacilli were obtained, cultures were washed with PBS, diluted into a series of concentrations, and incubated with DLF-1. The bacteria were plated for CFU enumeration. The fluorescent signal from labeled bacteria was measured with a multimode plate reader, using Cy5.5 and tdTomato wavelengths, respectively. Correlation between DLF-1 fluorescence and DLF-1 concentrations was analyzed first to determine dose-response relationship of DLF-1 labeling (Supplementary Figure 3A). The measured fluorescence increased as the concentration of DLF-1 went higher. Correlation between fluorescence and bacterial numbers was also analyzed using DLF-1 and tdTomato wavelengths, respectively (Figure 6A and Supplementary Figure 3B). Both DLF-1 specific fluorescence and tdTomato fluorescence correlated very well with bacterial CFUs. The threshold of detection for DLF-1 labeled dormant *Mtb* was determined as 3.1 × 10^5^ CFU (Figure 6B). DLF-1 labeling dormant *Mtb* was also confirmed with fluorescent microscopy using a deconvolution microscope as shown in Figure 6C for bacterial smear and in Figure 6D for intracellular dormant *Mtb* in THP-1 cells. The red pseudo color from DLF-1 co-localized with the green pseudo color from tdTomato very well both in smear slides and in cell infection slides, which confirms that DLF-1 is capable of labeling dormant *Mtb*, and the fluorescent signal can be measured when bacteria are inside of cells.

### DLF-1 can be applied to screen for *Mtb* genes critical for cell invasion *in vitro*

Conventionally, identification of *Mtb* virulence genes relies on comparison of bacterial CFUs between mutant and wild type strains under specific conditions. DLF-1 labeling has great potential to replace CFU enumeration *in vitro*. As DLF-1 labeling can quantitate labeled *Mtb* inside cells, we set out to identify bacterial genes that are critical for cell entry. Previous studies have reported that the heparin-binding hemagglutinin (HbhA) of *Mtb* plays an important role in infecting epithelial cells (Pethe et al., 2001; Mueller-Ortiz et al., 2002; Parra et al., 2004; Choi et al., 2013). HbhA was initially identified in *Mtb* and *M. bovis* BCG (Menozzi et al., 1996, 1998). It is located on the surface of the mycobacterium and mediates binding of the bacillus to epithelial cells and fibroblasts (Menozzi et al., 1996). Deletion of *hbhA (*Δ*hbhA)* leads to reduced adherence of bacteria to epithelial cells but shows no effect on adherence to macrophages (Pethe et al., 2001). We evaluated entry rates of Δ*hbhA* and the wild type Mt103 strain in A549 human lung epithelial cells using DLF-1 labeling, in comparison with CFU enumeration. Infection ratios of Δ*hbhA* to the wild type strain Mt103 at a MOI of 50 was <1 based on both DLF-1 fluorescent intensity and CFU results (Table 1). The infection ratio calculated using DLF-1 fluorescent intensity was consistent with the infection ratio calculated using CFU, which suggests that DLF-1 can be applied to screen bacterial genes that are critical for cell infection, and the Δ*hbhA* strain can serve as a positive strain for this purpose.

**Table 1:** **Bacterial cell infection ratios of mutants to the parental wild type strains calculated using DLF-1 labeling fluorescence and CFU, respectively**.

**Mutant/Mt**	**DLF-1^a^**	**CFU^b^**
Δ*hbh*A/Mt103	0.8 ± 0.1^*^	0.6 ± 0.3^*^
*plcC* mutant/CDC1551	0.7 ± 0.1^*^	0.5 ± 0.2^*^

*Bacterial infection ratio = entry rate of mutant strain/entry rate of wild type strain. Bacterial entry rate = intracellular bacterial fluorescence intensity or CFU measured after infection/total bacterial fluorescence intensity or CFU measured before infection*.

a *Infection ratios measured based on DLF-1 fluorescence*.

b *Infection ratios measured based on CFU*.

Values shown here represent the averaged ones from more than three independent experiments (

* *Wilcoxon-Mann-Whitney U-test showed that P < 0.05 for each ratio from independent experiments calculated by either DLF-1 or CFU based method)*.

Phospholipases are important bacterial virulence factors (Titball, 1998). Among them, phospholipase C (Plc) is important for replicating and cell-to-cell spread in mouse peritoneal macrophages for *Listeria monocytogenes* (Camilli et al., 1991). The Plc of *Clostridium perfringenes* is also named α toxin, which is haemolytic and suppresses neutrophil respiratory burst by disturbing host cell protein kinase C signaling (Terada et al., 1999). *Mtb* has four Plc genes, designated *plcA, B, C*, and *D*. The *plcA, B*, and *C* loci are clustered, while *plcD* is in a separated region in the chromosome of *Mtb* CDC1551. Expression of *plc* genes are upregulated at the 24-h post infection in macrophage infections, but deletions of *plc* genes do not impair growth in the human macrophage cell line THP-1. The *plcABC* triple mutant and *plcABCD* quadruple mutants are both attenuated in the late phase of mouse infection, indicating *plc* genes are important for *Mtb* survival in hosts (Raynaud et al., 2002).

We obtained the *plcC* mutant from BEI Resources (Manassas, VA) and examined its cell infection rate, in comparison to the infection rate of wild type CDC1551 in A549 cells. The bacterial infection ratio of *plcC* mutant to wide type CDC1551 was 0.7 ± 0.1 based on DLF-1 fluorescence intensity, and was 0.5 ± 0.2 based on CFU enumeration (Table 1). These results indicate that *plcC* is an important gene for *Mtb* entering alveolar epithelial cells. The detailed mechanism of how *plcC* helps bacilli infect epithelial cells remains to be explored.

## Discussion

Bacterial cell walls are organized with layers of PG located immediately outside of the cytoplasmic membranes (Scheffers and Pinho, 2005; Shih and Rothfield, 2006; Silhavy et al., 2010). PG is composed of a polysaccharide backbone along with alternating N-acetylmuramic acid and N-acetylglucosamine residues. These chains are then cross-linked via D-Ala-D-Ala motifs by transpeptidases (penicillin binding proteins; Scheffers and Pinho, 2005; Shih and Rothfield, 2006; Silhavy et al., 2010). The peptide cross links are essential for providing structural integrity and rigidity to the bacterial cell wall (Höltje, 1998; Shih and Rothfield, 2006). Almost all bacterial cell walls contain PG, but not all of them have the same structures. Gram-positive bacteria have a thicker PG layer than Gram-negative bacteria (Shockman and Barrett, 1983; Beveridge, 1999; Shih and Rothfield, 2006; Silhavy et al., 2010). Like Gram-negative bacteria, the PG cell wall layer of mycobacteria lies below an outer membrane, comprised of arabinogalactan (AG), and mycolic acids (MA), which are covalently linked together to form a MA-AG-PG complex (Hett and Rubin, 2008). This MA-AG-PG complex is the essential core of the mycobacterial cell wall and often the target of many drugs used to combat mycobacteria.

Glycopeptide antibiotics such as vancomycin, teicoplanin, and telavancin act by binding to the terminal peptide D-Ala-D-Ala of PG precursors, and are widely used in the treatment of infections caused by Gram-positive bacteria, especially MRSA (Reynolds, 1989; Finch and Eliopoulos, 2005; Pace and Yang, 2006; Butler et al., 2014). Thus, the DLF-1 probe could be used to label Gram-positive bacteria. Vancomycin and its analogs can bind to the mycobacterial cell wall components PG and/or lipid II (MIC of vancomycin against *Mtb*: 2 μg/ml) (Lambert, 2002; Dinesh et al., 2013; Soetaert et al., 2015). To tag vancomycin, fluorescent molecules must be introduced at sites that do not interfere with its ability to bind to PG precursors. Vancomycin has two amine groups that are amenable to chemical modification. The amine located on the disaccharide is more chemically reactive (Kahne et al., 2005), and as the disaccharide is not involved in D-Ala-D-Ala binding, we attached Cy5.5 to this amine group to produce our probe, DLF-1. We verified that DLF-1 has a high affinity for D-Ala-D-Ala independently using isothermal titration calorimetry (ITC). Because this novel fluorescent labeling probe has vancomycin as an anchor, it is critical to confirm that DLF-1 does not affect bacterial growth at the working concentration for imaging. We have verified that 100 nM of DLF-1 does not affect *Mtb* growth.

Fluorescently labeled glycopeptide antibiotics have been used: (1) to detect bacterial infections *in vivo* (van Oosten et al., 2013), (2) to study the genes that control the biosynthesis of peptidoglycan (Tiyanont et al., 2006), and (3) to investigate cell wall synthesis in *B. subtilis* (Daniel and Errington, 2003). Recently, a study demonstrated that a fluorescent dye IR800CW conjugated with vancomycin can be applied to image *Staphylococcus aureus* infection in mice with a high specificity for detection of Gram-positive bacterial infections (van Oosten et al., 2013). We explored the application of the DLF-1 in detecting active replicating and dormant *Mtb*, and found that DLF-1 can effectively label both actively replicating and dormant *Mtb*. Furthermore, we developed an approach to use DLF-1 labeled *Mtb* to quickly screen for bacterial genes that are important for infectious and nonphagocytic cells. Because the DLF-1 imaging method does not require a recombinant strain, it can be directly applied to an established mutant library to study bacterial genes *in vitro*. This application of DLF-1 has been verified by using the Δ*hbhA* mutant of Mt103 strain. It is well-known that HbhA is critical for *Mtb* infection of epithelial cells (Pethe et al., 2001; Mueller-Ortiz et al., 2002; Parra et al., 2004; Choi et al., 2013). CFU enumeration results confirm that deletion of the *hbhA* gene did impair bacterial infection in A549 cells, as reported previously (Pethe et al., 2001). Using Δ*hbhA* as a positive control, we have found by DLF-1 labeling and CFU enumeration that another gene of *Mtb, plcC*, is also important for bacterial entry of epithelial cells. To our knowledge, this is the first report showing that *plcC* plays an important role for infecting epithelial cells. The fact of mutant of *hbhA* or *plcC* had reduced entry rates but was still able to enter epithelial cells indicates that *Mtb* has other genes that compensate function of HbhA or PlcC. As lung epithelial cells are the first barriers to *Mtb* infection, how *Mtb* enters epithelial cells is very critical for establishment of lung infection at earlier stages. It remains to further explore the detailed mechanism of *plcC* in lung infection and if the other three *plc* genes of *Mtb* share similar function.

The novel fluorescent imaging method described here provides a useful substitute for traditional time-consuming, labor-intensive approaches of quantitating bacterial numbers in TB research *in vitro*. However, at current stage, DLF-1 cannot be directly applied to TB diagnostic assays as it is not a mycobacterial specific probe. It remains to be investigated if DLF-1 is capable of *in vivo* imaging of *Mtb* infection in live animal models and *ex vivo* imaging of infected organs. The dye Cy5.5 on DLF-1 makes it ideal for *in vivo* imaging, as its excitation and emission wavelengths are within the “near infrared window,” in which the absorbance coefficients of hemoglobin water and hemoglobin have less absorbance, so excitation light can penetrate through the animal without significant loss (Weissleder, 2001). In the future, we will validate the sensitivity, reproducibility and accuracy of DLF-1, by quantitating bacterial numbers in *Mtb* infection *in vivo*. We will also evaluate application of this imaging technique to quantification of drug response in anti-TB therapy. As DLF-1 labels both viable bacteria and dead ones, it can be utilized together with other techniques that measure viable bacteria only, such as fluorescence protein expressing strains, to evaluate bactericidal activities in laboratory. The dead bacterial number could be calculated as the number of bacteria estimated by DLF-1 labeling subtracting the number of bacteria estimated by measuring fluorescent intensity of the fluorescence protein expressing strain. In summary, this strategy could be applied to preclinical screening therapies in a more time-efficient manner, and be used to real-time quantitate bacterial load while studying bacterial genes and host immune factors involved in active TB or LTBI. The success of these studies would accelerate screening for anti-TB therapy in animal models and enhance studies of bacterial and host factors in TB.

## Author contributions

Conceived and designed the experiments: DY, FD, KM, RL, MK, and YK. Performed the experiments: DY, FD, KM, and YK. Analyzed the data: DY, FD, KM, RL, MK, and YK. Contributed reagents/materials/analysis tools: RL, MK, and YK. Wrote the manuscript: DY, FD, and YK.

## Funding

This work was supported by the National Heart, Lung, And Blood Institute of the National Institutes of Health under award number R21HL115463, University of Tennessee Health Science Center start-up funding, and the American Lebanese Syrian Associated Charities, St. Jude Children's Research Hospital.

### Conflict of interest statement

The authors declare that the research was conducted in the absence of any commercial or financial relationships that could be construed as a potential conflict of interest.

## Abbreviations

TB: tuberculosis
*Mtb*: *Mycobacterium tuberculosis*
CFU: colony forming unit
ITC: isothermal titration calorimetry
PBS: Phosphate buffered saline
MOI: Multiplicity of infection
PG: peptidoglycan
AG': arabinogalactan
MA: Mycolic acid
D-Ala-D-Ala: D-alanyl-D-alanine.
