# Supplementary material for: A Fluorescent Probe for Detecting Mycobacterium tuberculosis and Identifying Genes Critical for Cell Entry
Source: Front Microbiol. 2016 Dec 20;7:2021. doi: 10.3389/fmicb.2016.02021 (PMC5168438; doi:10.3389/fmicb.2016.02021)
Supplement: Supplementary file 1 [file Presentation1.PDF]

SUPPLEMENTARY FIGURE LEGENDS

Supplementary Fig. 1. Correlation between OD600 measured with the Tecan Infinite 200 Pro multimode reader and OD600 measured with the Genesys 20 spectrometer. Cultures of *M. smegmatis* (**A**), *M. bovis BCG* (**B**), and *Mtb* (**C**) were measured optical density (OD) with the commonly used Genesys 20 spectrometer at absorbance 600 nm, and adjusted to OD=1. The bacteria were diluted into OD=0.5, 0.25 and 0.1. Three samples from OD=1 and each dilution were loaded into a 96-well plate and measured OD values with the Tecan Infinite 200 Pro multimode reader at absorbance 600 nm. The average OD values at each dilution measured with the Tecan reader were plotted against the OD values measured with the Genesys 20.

Supplementary Fig. 2. **Differentiation of DLF-1 labeled *Mtb from non-labeled Mtb***. 10^3-10^6 CFU of *Mtb* expressing tdTomato were incubated with 100 nM DLF-1 in PBS for 1 h. Fluorescence labeled DLF-1 on bacteria were measured after removing the extra probes by centrifugation, with gain 150, ex. 670nm & em. 700nm (Cy5.5) (**A**), and with gain 100, ex.550 & em.590 (tdTomato) (**B**). Measured fluorescence was converted into log scale for Y-axis. Student's T-test was performed to analyze fluorescence differences between DLF-1 labeled and non-labeled groups at each CFU category. Error bars represent standard deviations of four samples. One-way ANOVA test was conducted for assessing overall differences among groups, and Turkey's multiple comparison tests were applied to assess differences between two groups. * $P<0.05$; ** $P<0.01$; and *** $P<0.001$.

Supplementary Fig. 3. Correlation between fluorescence intensity of DLF-1 and concentration of DLF-1 (A); **and correlation between fluorescence intensity of tdTomato wavelengths (squares) and bacterial numbers of dormant *Mtb*** (**B**). Dormant tdTomato-expressing *Mtb* was incubated with various concentrations of DLF-1, and fluorescence intensities were measured with DLF-1 and tdTomato wavelengths, respectively.

Supplementary Table 1. Thermodynamic parameters for binding of peptide AcKAA with vancomycin or DLF-1

	Vancomycin			DLF-1		
[AcKAA] (syringe), µM	**600**			**100**		
[compound] (cell), µM	**32**			**7**		
**ΔG, cal/mol**	-7610.71	-7628.85	-7647.19	-7676.27	-7703.88	-7723.62
**ΔH, cal/mol**	-12560.00	-13890.00	-13640.00	-5786.00	-6234.00	-6051.00
**-TΔS, cal/mol**	4949.29	6261.15	5992.82	-1890.27	-1469.88	-1672.62
Temp, in Kelvin	298.15	298.15	298.15	298.15	298.15	298.15
ΔS, cal	-16.6	-21	-20.1	6.34	4.93	5.61
K_a	3.86E+05	3.92E+05	4.08E+05	4.23E+05	4.44E+05	4.59E+05
K_d	2.59E-06	2.55E-06	2.45E-06	2.36E-06	2.25E-06	2.18E-06
N (stoichiometry)	1.06	0.989	0.966	0.982	0.986	0.995
**ΔG, kcal/mol**	-7.61071	-7.62885	-7.64719	-7.676271	-7.70388	-7.72362
**ΔG, kcal/mol error**	0.02			0.02		
**ΔH, kcal/mol**	-12.56	-13.89	-13.64	-5.786	-6.234	-6.051
**ΔH, kcal/mol error**	0.71			0.23		
**-TΔS, kcal/mol**	4.94929	6.26115	5.992815	-1.890271	-1.46988	-1.67262
**-TΔS, kcal/mol error**	0.69			0.21		
**average K_a**	3.95E+05			4.42E+05		
**SD of K_a**	1.14E+04			1.81E+04		
**average K_d**	2.53E-06			2.26E-06		
**SD of K_d**	7.20E-08			9.34E-08		
**%CV of K_d**	2.84			4.12		
**average N**	1.01			0.99		
	**Vancomycin**			**DLF-1**		
**average ΔG, kcal/mol**	-7.63			-7.70		
**average ΔH, kcal/mol**	-13.36			-6.02		
**average -TΔS, kcal/mol**	5.73			-1.68		

Supplementary Table 2. Drug tolerance of dormant *Mtb* in the Wayne hypoxia model in comparison with actively replicating *Mtb*

	Dormant *Mtb*		Active Replicating *Mtb*	
	With RIF (0.1 µg/ml; n=6	No RIF (n=3)	With RIF (0.1 µg/ml; n=6	No RIF (n=3)
Average CFU/ml	$4.7X10^{6}$	$2.0X10^{7}$	$1.3X10^{7}$	$4.9X10^{8}$
Rate of tolerance ±95% confidence interval	23.5±4.8		2.7±0.5	
