# Supplementary figures and images for: A Fluorescent Probe for Detecting Mycobacterium tuberculosis and Identifying Genes Critical for Cell Entry
Source: Front Microbiol. 2016 Dec 20;7:2021. doi: 10.3389/fmicb.2016.02021 (PMC5168438; doi:10.3389/fmicb.2016.02021)

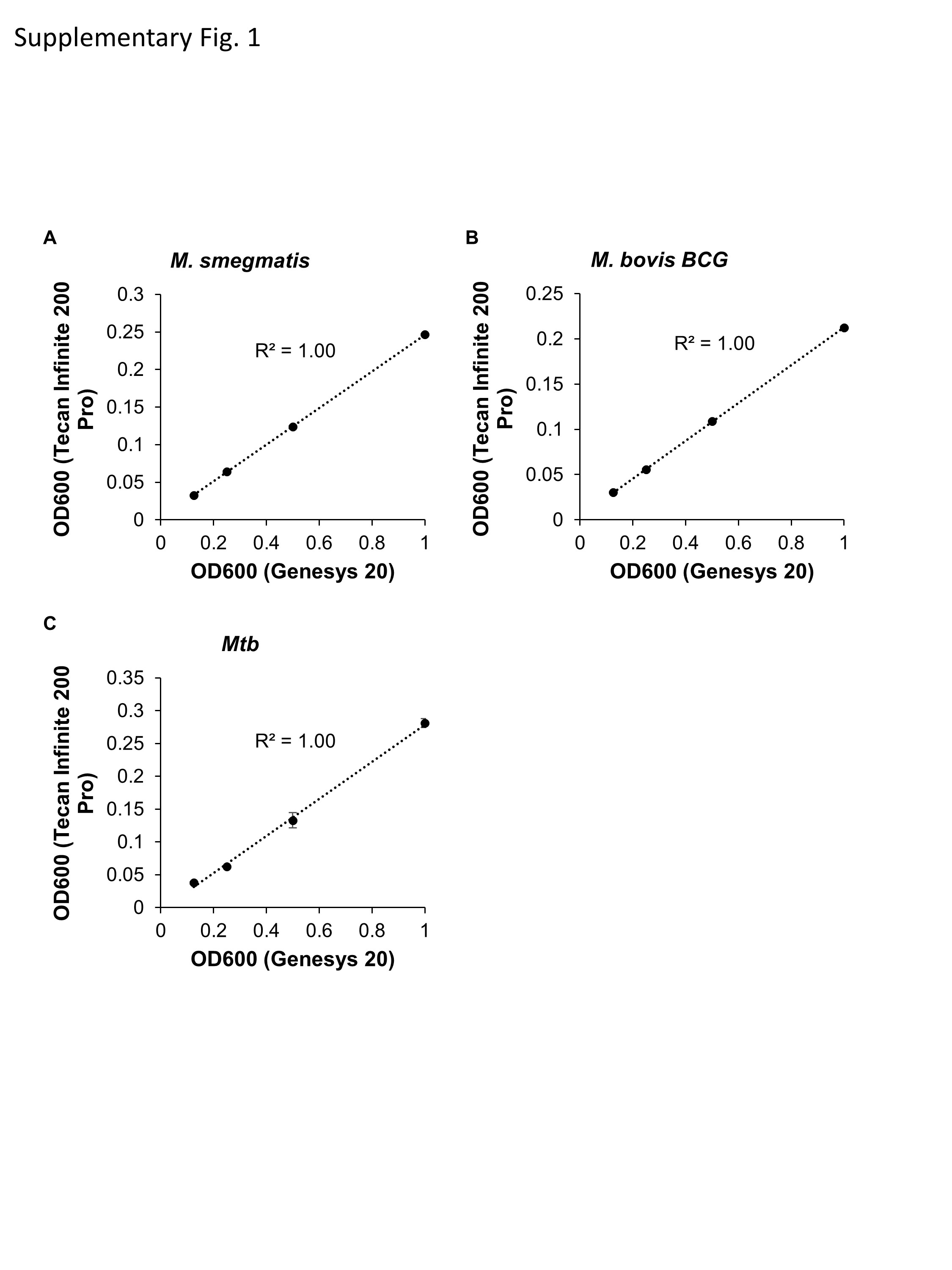

Supplement: Supplementary file 2 [file Image1.JPEG]

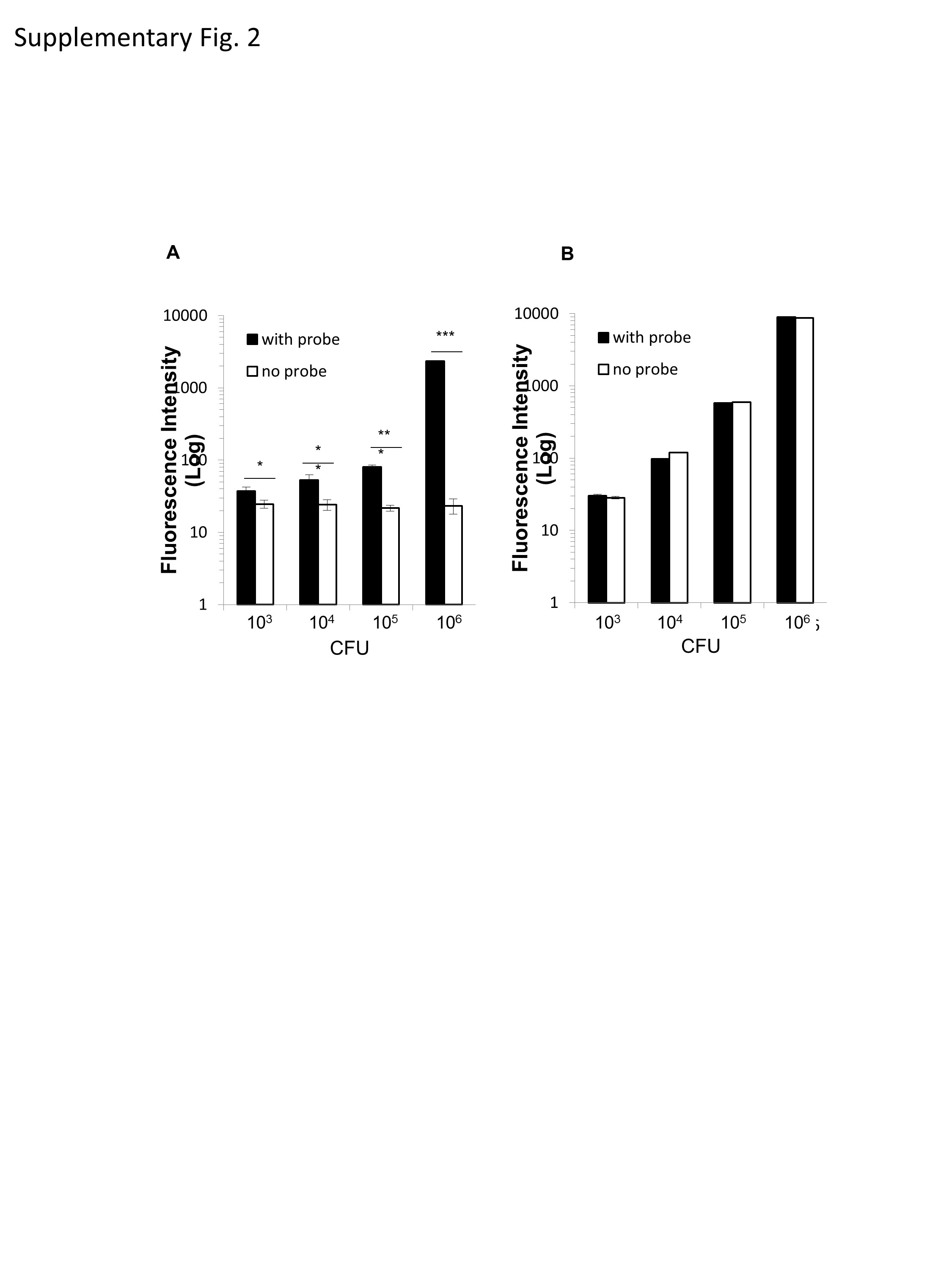

Supplement: Supplementary file 3 [file Image2.JPEG]

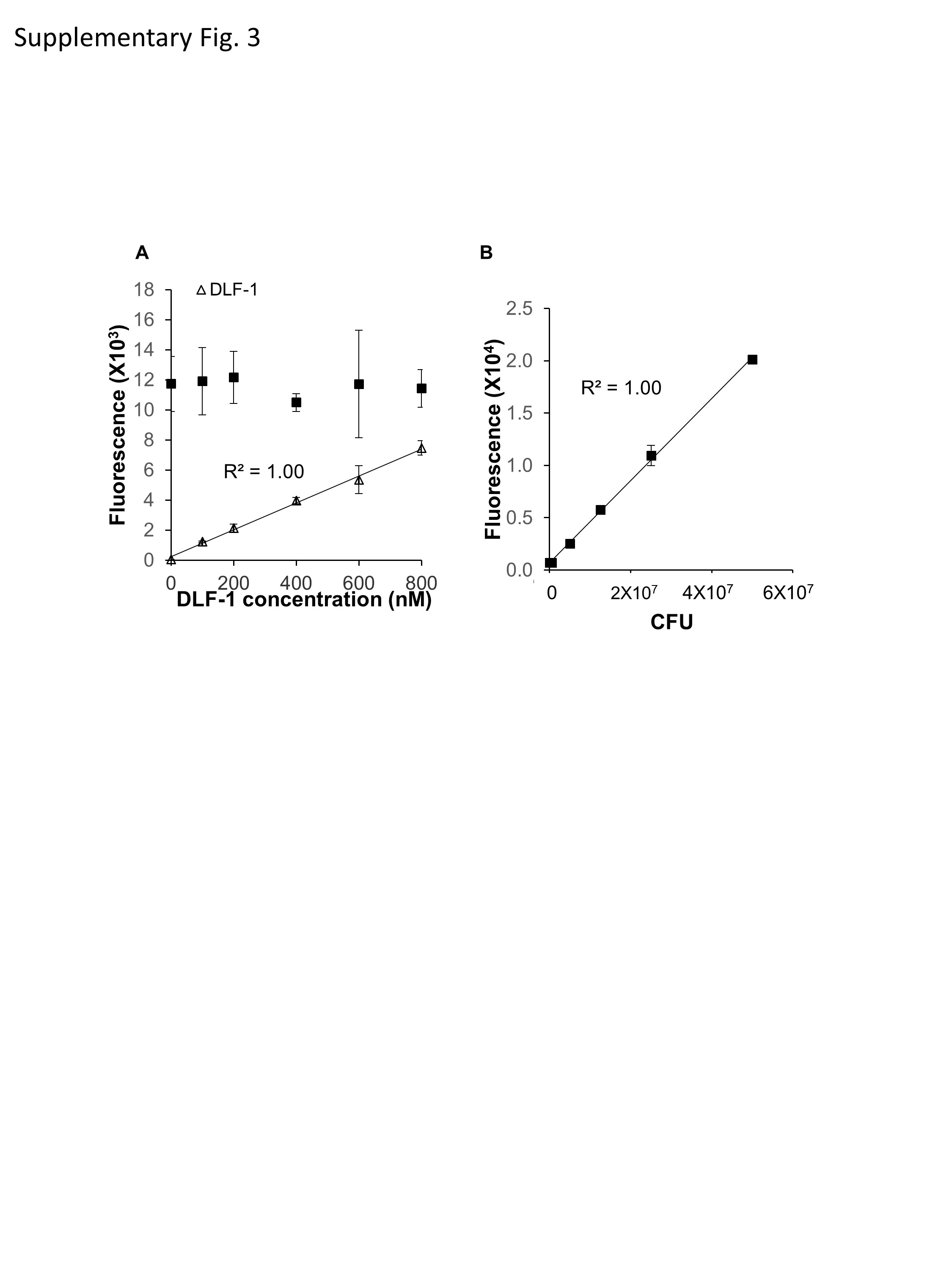

Supplement: Supplementary file 4 [file Image3.JPEG]
